# Light Color Influences Incubation Characteristics, Postnatal Growth, and Stress Physiology with a Lack of Expression Changes of Myf5 and Myf6 Genes in Gerze Native Chicken

**DOI:** 10.3390/ani15162347

**Published:** 2025-08-11

**Authors:** Godswill Arinzechukwu Iwuchukwu, Uğur Şen, Hasan Önder, Elif Cilavdaroğlu, Umut Sami Yamak

**Affiliations:** 1 Department of Agricultural Biotechnology, Faculty of Agriculture, Ondokuz Mayis University, 55139 Samsun, Türkiye; godswill2014.gi@gmail.com; 2 Department of Animal Science, Faculty of Agriculture, Ondokuz Mayis University, 55139 Samsun, Türkiye; honder@omu.edu.tr (H.Ö.); elif.cilavdaroglu@omu.edu.tr (E.C.); usyamak@omu.edu.tr (U.S.Y.)

**Keywords:** Gerze chicken, light color, hatching characteristics, growth performance, stress response, myogenic regulatory factors

## Abstract

Light exposure during incubation can influence hatching characteristics, post-hatch growth, and physiological responses in poultry. This study aimed to evaluate the impact of pre-development exposure to different cold-colored light emitting diode (LED) lights on incubation characteristics, growth performance, stress physiology, and myogenic regulatory factors (MRFs) expression level in the slow-growing native Gerze chicken breed. Fertilized eggs were incubated under red, green, white, or dark conditions. The shortest hatch window was observed under red light (42 h), while the dark condition resulted in the longest (84 h) (*p* < 0.05). White light exposure reduced hatchability compared to the other groups (*p* < 0.05), whereas green and red lights enhanced both chick weight at hatch and egg-to-chick conversion efficiency (*p* < 0.001). Chicks incubated under green light exhibited superior body weight during the first four weeks (*p* < 0.05), but those from the red light group maintained consistently higher weights thereafter (*p* < 0.05). The highest serotonin levels were detected in chicks from the dark group, while the lowest were associated with green light exposure. Red light exposure was associated with the lowest corticosterone concentrations (*p* < 0.05). Gene expression analysis revealed no significant differences in MRFs across groups. In conclusion, the spectral level of light during incubation exerts measurable effects on hatch dynamics, post-hatch growth, and hormonal regulation in Gerze chicken.

## Simple Summary

In this day and age, where sustainable animal production and animal welfare are becoming increasingly important, scientists are exploring how the external environment of animals can impact their health and growth. This study investigates how exposing chicken embryos to differently colored light during incubation affects their development after hatching. Light color during incubation has been shown to reduce stress and fear responses in chicks, which may lead to healthier and more resilient animals. In the same vein, previous studies also suggest that certain light colors, such as green, may help chicks grow faster in terms of muscles development and expression of muscle related genes. In this research, we focus on a native chicken breed and aim to understand whether different light colors during incubation influence the genes that control muscle growth and stress hormones. Specifically, we look at two genes known to affect muscle development and examine levels of hormones related to stress and well-being in the chicks. The findings from this study could help conserve this local chicken breed and assist farmers in raising healthier poultry in a more natural and stress-free way, supporting both animal welfare and more sustainable poultry farming practices.

## 1. Introduction

The incubation environment plays a pivotal role in determining hatchability and embryonic development in poultry species. Environmental variables such as temperature, humidity, egg turning frequency, gas concentrations (CO_2_ and O_2_), and lighting conditions within incubators are among the most thoroughly examined factors in poultry science [1]. Beyond their immediate effects on embryogenesis, changes in incubation conditions can influence physiological responses and behavior in birds’ post-hatch.

Among these variables, lighting—particularly its presence, duration, and spectral composition—has gained increasing attention. Studies have demonstrated that light exposure during incubation may positively influence post-hatch welfare by reducing fear responses and improving stress resilience [2,3,4,5]. Light treatments have also been associated with modifications in hormonal profiles, such as corticosterone and serotonin levels, suggesting their role in shaping stress physiology during early development [6,7] and behavioral fear responses to environmental stimuli [4,8,9]. However, while the physiological implications of light exposure have been explored [3,4,10,11,12,13], there is a relative paucity of research on how specific light wavelengths affect both hatching dynamics and post-hatch performance, particularly in native chicken breeds.

The MRF gene family—including myogenic factor 5 (Myf5) and myogenic factor 6 (Myf6)—is known to play a critical role in embryonic and postnatal skeletal muscle development, thus influencing growth and meat yield [14]. Hluchý et al. [15] reported that different wavelengths of light can exert varying effects on the embryonic development process in chickens. In a separate study, Shafey and Al-Mohsen [11] demonstrated that exposing embryos to green light at an intensity of 1340–1730 lux from day 5 to day 15 of incubation significantly enhanced their growth. Tong et al. [16] also reported that the use of monochromatic green LED light during the first 18 days of incubation reduced the total incubation time by 3.4 h. Huth and Archer [17] found that exposing broiler eggs to white LED light throughout the entire incubation period improved hatchability. However, the same exposure had no significant effect on the hatchability of layer eggs. This discrepancy was attributed to differences in eggshell pigmentation, which may cause unequal light filtration between broiler and layer eggs. Similarly, Hluchý et al. [15] observed that exposing chicken eggs to red light during incubation resulted in a higher hatch success rate compared to blue light, with white light yielding the highest hatchability overall. Archer [7] further noted that while red light improved hatchability in white-shelled layer eggs, it had no effect on the hatchability of broiler eggs. Research has also shown that exposing chicken [18,19] and turkey embryos [20] to green light during incubation promotes greater development of breast muscle in chicks. Halevy [21] demonstrated that green light exposure enhances growth by promoting the coordination of muscle fibers as well as the proliferation and differentiation of mature muscle cells. Additionally, Zhang et al. [22,23] found that green light exposure during incubation led to increased proliferation and differentiation of satellite cells and improved feed conversion efficiency throughout the rearing period.

**Figure 1:**
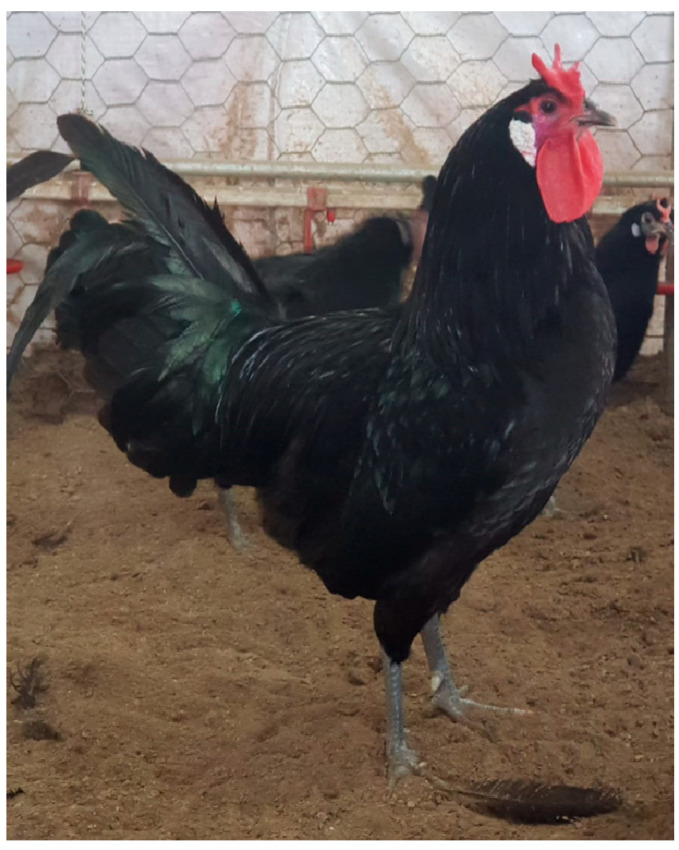
A picture showing Gerze chicken.

Although phenotypic variability in growth and production traits has been documented in Turkey’s native Gerze chicken breed [24,25,26], the underlying genetic or environmental mechanisms driving this diversity remain insufficiently characterized. Given the growing importance of preserving and optimizing native genetic resources in the face of environmental uncertainties and global changes in animal production systems, the Gerze breed (Figure 1) presents a valuable model. Its potential resilience to harsh conditions, combined with consumer interest in the unique quality of native breed products, underscores the need for targeted studies that enhance its productivity without compromising adaptability. This study is particularly significant as it is the first to investigate the expression levels of muscle fiber development genes in the Gerze breed, addressing a critical gap in the literature. Understanding the meat production capacity of local breeds can enhance their competitiveness against commercial lines and support the preservation of genetic diversity. Also, in accordance with sustainability in animal production, conserving and enhancing native genetic resources will be vital, especially for adaptability to changing environments.

As current local breeds may lack high economic productivity, it is difficult to predict their future demand. However, due to anticipated declines in crop-based feed production and the adverse effects of global warming, there may be a shift toward raising native breeds that are more resilient to harsh conditions. In such a scenario, native breeds adapted to local environments may become essential for sustainable livestock production. These genetic resources also play an important role in education, research, and preserving ecological balance. Moreover, products derived from native breeds often possess unique flavors and qualities, which are increasingly preferred in some countries.

As one of Türkiye’s two native chicken breeds, improving the growth and productivity of Gerze chickens can contribute to the conservation of this genetic resource and reduce reliance on imported breeding stock. In this context, the study investigates whether different colored LED light treatments during incubation can create epigenetic effects that influence growth performance and stress tolerance in Gerze chicks, which have been selectively bred for better weight gain despite their slow-growing nature.

This study investigates the effects of cold-colored LED light treatments (white, red, and green) and darkness during incubation on hatchability, post-hatch growth performance, stress-related hormonal responses, and the expression levels of Myf5 and Myf6 genes in Gerze chickens. By evaluating both physiological and molecular outcomes, this work aims to provide novel insights into optimizing incubation protocols to support the sustainable utilization of slow-growing native poultry genetic resources.

## 2. Materials and Methods

### 2.1. Animal Material

This study was conducted at the Poultry Breeding Unit of the Animal Science Department, Faculty of Agriculture, Ondokuz Mayıs University. Fertilized eggs obtained from 38–40-week-old Gerze breed hens were used as the study material. The eggs selected for the experiment weighed between 45–50 g and exhibited no deformities. All eggs were collected over a period of one week and stored under controlled conditions of 18 °C and 60% relative humidity. Eggs from each day were grouped separately. After the storage period, the eggs were weighed to determine the storage weight loss prior to incubation Equation (1).

Storage loss (%) = [(Initial egg weight − Egg weight after storage)/Egg weight after storage] × 100(1)

### 2.2. Incubation Conditions

This study employed the same brand and model for both incubation (Çimuka T2400S, Çimuka Hatchery Industry and Trade Ltd. Co., Ankara, Türkiye) and hatching (Çimuka T2400H, Çimuka Hatchery Industry and Trade Ltd. Co., Ankara, Türkiye) machines. A randomized design was used, with one incubator set under standard dark incubation conditions and the others equipped with cold white (YuLED Silicone Strip LED 220 V, HBCV0000023KPV, Wavelength: 6000 K–6500 K), red (YuLED Silicone Strip LED 220 V, HBCV000006R87Z, Wavelength: 610–625 nm), or green (YuLED Silicone Strip LED 220 V; HBCV000006R885, Wavelength: 515–525 nm) LED strips. Eggs were transferred to incubators and subjected to standard incubation conditions (37.5 °C temperature and 55% relative humidity) for an 18-day pre-development period under different LED light treatments (white, red, green) or complete darkness. After the 18th day of incubation, eggs were transferred to hatchers under standard hatching conditions (36.5 °C temperature and 65% relative humidity) with no light exposure and complete darkness. The observation windows of the incubators and hatchers were insulated to prevent external light interference. Temperature and humidity in both the incubators and hatchers were monitored and controlled using digital thermo-hygrometer data loggers (CEM DT-172, CEM Instruments, Shenzhen, China). A total of 114 eggs were used per treatment group, except for the dark control group, which included 108 eggs. Eighty chicks were randomly selected from the hatched chicks for each treatment group.

### 2.3. Post-Hatch Procedures

At hatching, all chicks from each treatment group were weighed and evaluated for quality based on the Pasgar score criteria [27] presented in Table 1. The highest possible Pasgar score is 10, with points deducted for abnormalities across five categories as outlined.

At the end of the hatching period, unhatched eggs were broken to determine the number of infertile eggs. Among the fertile eggs, the number of embryos that died at early (0–7 days), middle (8–17 days), and late (18–21 days) stages were recorded. These data were used to calculate hatchability Equation (2), fertility Equation (3), incubation efficiency Equation (4), egg-to-chick conversion Equation (5), and embryo mortality rates Equation (6).

Hatchability (%) = [(Live chicks)/(Fertile eggs)] × 100(2)

Fertility (%) = [(Fertile eggs)/(Total eggs)] × 100(3)

Incubation Efficiency (%) = [(Live chicks)/(Total eggs)] × 100(4)

Egg-to-Chick Conversion (%) = [(Chick weight)/(Egg weight)] × 100(5)

Embryo Mortality (%) = [(Dead embryos)/(Fertile eggs)] × 100(6)

### 2.4. Chick Start and Grow-Out Period

Due to the slow growth rate of the Gerze breed, the chicks were subjected to a total feeding period of 14 weeks, including a 6-week starter phase and an 8-week grow-out phase. After hatching, healthy chicks from each treatment group were randomly placed in groups of 40 in five-tiered poultry cages (Çimuka, Chick Cage CB25, Çimuka Hatchery Industry and Trade Ltd. Co., Ankara, Türkiye) equipped with a nipple drinking system and feeders. The chicks were reared in these cages at approximately 35 °C for the first three weeks. From 0 to 3 weeks of age, chicks were fed a commercial starter feed (19% crude protein, 2900 kcal/kg metabolizable energy) ad libitum. At 3 weeks of age, 80 chicks from each treatment group were randomly selected, wing-banded, and weighed. After this, chicks were transferred to pens measuring 3 × 3 m with infrared heaters, two feeders, and an automatic nipple drinker system. Each treatment group was randomly assigned to pens within the housing unit. The bedding material used in the cages was coarse wood shavings. During the grow-out period, temperatures were maintained at 26–28 °C from 3–6 weeks of age and at 24–26 °C for the remaining period. The lighting schedule included 24 h of light (20 lux) with 0 h dark period for the initial 3 days, followed by an intermittent lighting program of 20 h of light with 4 h of darkness from day 3 to week 14 until the study’s conclusion. Chicks were vaccinated for Gumboro on day 13 and Newcastle disease and infectious bronchitis on day 50 of the trial. Regular monitoring also ensured the birds’ health status, and bird weights were recorded fortnightly.

### 2.5. Slaughter and Muscle Sampling

At the end of the feeding period, all chicks were fasted for 12 h, although water was provided ad libitum. Live weights were recorded according to regulatory standards. Following weighing, 10 male and 10 female chicks from each treatment group were randomly selected for slaughter. The right and left pectoralis major (PM) and gastrocnemius (GN) muscles were isolated from the carcasses, cleaned of fat and connective tissue, and 2 × 5 × 2 cm samples were obtained from the right side of the carcass. The tissue samples were frozen in liquid nitrogen and stored at −80 °C until RNA analysis.

### 2.6. Plasma Serotonin Evaluation

Blood samples were collected into heparinized tubes before slaughter. Plasma was separated by centrifugation at 5000× *g* for 30 min at 4 °C and stored at −20 °C for serotonin analysis. Plasma serotonin levels were measured using a commercially available ELISA kit (5-HT BT LAB Chicken Serotonin ELISA Kit, analytical range 0.5–150 ng/mL), following the manufacturer’s instructions. Absorbance was measured at 450 nm using a microplate reader (BMG SPECTROstar Nano, BMG LABTECH, Ortenberg, Germany).

### 2.7. Plasma Corticosterone Evaluation

Plasma corticosterone levels were quantified using a commercially available ELISA kit (detection range 0.1–40 ng/mL, Chicken Corticosterone, CORT ELISA Kit, BT LAB, Jiaxing, China). The assay followed the manufacturer’s protocol, with measurements taken at 450 nm using a microplate reader (BMG SPECTROstar Nano, BMG LABTECH, Ortenberg, Germany).

### 2.8. Total RNA Isolation

Total RNA was isolated from the PM and GN muscles using Trizol reagent (BioBasic, Toronto, ON, Canada). The tissue samples were homogenized in liquid nitrogen, followed by the addition of 1 mL of Trizol reagent. After mixing and incubation, RNA was extracted by the addition of chloroform, followed by centrifugation. The resulting aqueous phase was collected, and RNA was precipitated with isopropanol. After RNA isolation, 5 µL DNase I (2U/µL) treatment was performed to remove any DNA contamination from the samples. Following ethanol washing, RNA pellet was re-suspended in RNase-free water. RNA concentration and purity were measured using a NanoDrop 2000 spectrophotometer (Thermo Fisher Scientific Inc., Waltham, MA, USA), and samples were used only if the A260/A280 and A260/A230 ratios were between 2.0 and 2.2.

### 2.9. Complementary DNA (cDNA) Synthesis

RNA samples stored at −80 °C were diluted to 1 µg/µL for cDNA synthesis. A commercial cDNA kit (BIORAD iScript cDNA, 1708890, Hercules, CA, USA) and a Thermal Cycler (BIORAD, Hercules, CA, USA) device were used for the cDNA synthesis. The analysis was carried out according to the instructions provided by the manufacturer of the commercial kit. For cDNA formation, the RNA amount was measured for each sample and placed in PCR tubes. Then, nuclease-free water, 4 µL of 5× iScript reaction mix, and 1 µL of iScript Reverse Transcriptase were added, and the final volume was adjusted to 20 µL. The PCR tubes containing the samples were placed in the Thermal Cycler (BIORAD, Hercules, CA, USA) for cDNA synthesis. The integrity and presence of cDNA were confirmed with a 1.5% agarose gel. The obtained cDNA samples were stored at −20 °C until quantitative real-time polymerase chain reaction (qRT-PCR).

### 2.10. Quantitative Real-Time PCR (qRT-PCR) Analysis

The expression levels of the Mfy5 and Mfy6 genes in PM and GN muscle samples were determined by quantitative real-time polymerase chain reaction (qRT-PCR). Synthetic oligonucleotide primers designed according to the relevant gene regions were used, and GAPDH was employed as the reference gene (housekeeping gene). Primers and the reference gene were dissolved in nuclease-free water to create 100 µM stock solutions. A total of 50 µL of each stock primer was taken and diluted to a final volume of 950 µL with nuclease-free water to prepare 1 mL of 10 µM primers for real-time PCR. The sequences of the primers for the Mfy5 and Mfy6 genes and the reference gene are shown in Table 2. The PCR protocol used was as follows: initial denaturation at 95 °C for 30 s, denaturation at 95 °C for 5 s, primer binding at 60 °C for 30 s, and extension at 65 °C for 5 s. All procedures were repeated at least three times for reliability. To calculate qRT-PCR efficiency, standards of different concentrations (1, 1/10, 1/100, and 1/1000) were used. The qRT-PCR efficiency for the study was between 1.60 and 2.10. The expression levels of Mfy5 and Mfy6 were normalized against the reference genes and analyzed using the 2^−∆∆Ct^ method.

### 2.11. Statistical Analysis

The data obtained throughout the study were analyzed using the SPSS 20.0 package program licensed by Ondokuz Mayıs University. Only the effect of light treatment during the incubation period was used as the explanatory variable in this study. Sex was used as a co-factor, and the effects on growth performance and other parameters were eliminated. The normality of the data was tested using the Shapiro–Wilk test, and it was determined that the data followed a normal distribution (*p* > 0.05). The homogeneity of variances was evaluated using the Levene test, and it was determined that the variances were homogeneous. Under these conditions, one-way analysis of variance (ANOVA) was used to evaluate the data, and the Duncan multiple comparison test was applied to compare the means. The Kruskal–Wallis H test was used for the Pasgar score. Chi-square goodness-of-fit analysis was used to analyze the percentage-based measurements. Pearson correlation analysis was used to determine the relationship between growth characteristics and the expression levels of Mfy5 and Mfy6 genes.

## 3. Results

### 3.1. Pre-Incubation Characteristics

The number of eggs used in each treatment group, the average initial weight, the weight at transfer, and storage loss are presented in Table 3. During the pre-incubation period, no statistically significant differences were found between the treatment groups (green, red, white cold LED light, and darkness) in terms of the number of eggs, initial egg weight, weight at transfer, or weight loss during storage (*p* > 0.05).

### 3.2. Hatch Window and Incubation Efficiency

**Figure 2:**
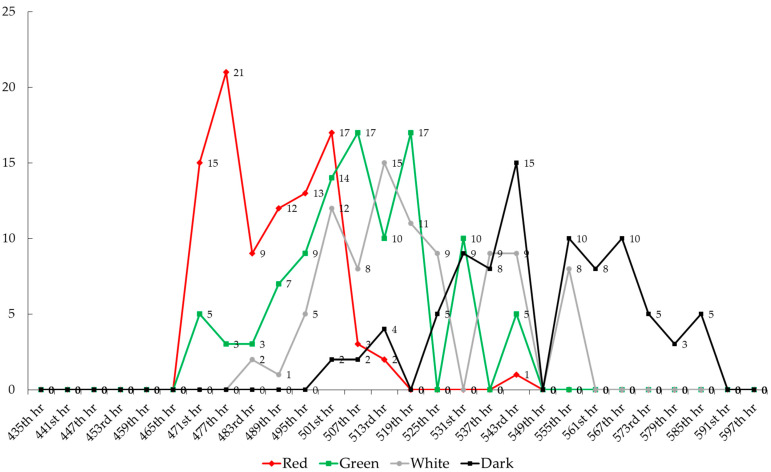
Distribution of the number of chicks according to the pre-development and post-hatching hatching intervals of eggs in the treatment groups.

The distribution of chick hatch times from eggs exposed to green, red, white cold LED light, and darkness during the pre-incubation period is shown in Figure 2. In eggs subjected to red LED light during pre-incubation, hatching began at hour 471 and ended at hour 513, following 432 h of incubation. The hatch window (total hatching time) for the red light group was calculated as 42 h. In the green LED light group, hatching started at 471th h and ended at 543rd h, resulting in a hatch window of 72 h. For the white LED light group, chick hatching started at hour 483 and ended at hour 555, also with a 72-h hatch window. In the dark group, hatching began at hour 501 and ended at hour 585, with an 84-h hatch window. It was determined that the red LED light shortened the hatch window, while darkness prolonged it (*p* < 0.05).

### 3.3. Chick Quality and Hatchability Parameters

Table 4 presents the incubation traits of Gerze eggs exposed to green, red, and white cold LED light and darkness during pre-incubation, as well as chick weight, hatchability of fertile eggs, and average Pasgar scores post-hatch. According to statistical analysis, the interaction between group and sex was not significant for live weight and weight gain at various ages (*p* > 0.05); therefore, only the effects of light treatment are shown. Since male and female chicks showed similar hatch weights and hatchability across all groups, these averages are not separately presented. No statistically significant differences were found in hatchability of fertile eggs, fertility rate, eggshell death, chick mortality, early embryonic death, mid-embryonic death, late embryonic death, total embryonic mortality, or Pasgar score among the treatment groups (*p* > 0.05). However, light color during pre-incubation significantly affected hatchability (*p* < 0.05). The white LED light significantly reduced hatchability compared to other LED lights and darkness (*p* < 0.05). Furthermore, green and red LED lights significantly increased chick weight at hatch compared to the dark control and white LED light (*p* < 0.001). Similarly, the hatchability of fertile eggs was significantly higher in green and red LED groups compared to the white LED and dark groups (*p* < 0.001).

### 3.4. Post-Hatch Growth Performance

Table 5 shows the live weights and live weight gains of Gerze chicks at various ages from eggs exposed to green, red, and white cold LED light and darkness during pre-incubation. Statistical analysis indicated that the group × sex interaction was not significant (*p* > 0.05), so only the effects of light treatment are shown. Since male chicks in all treatment groups had significantly higher live weights and weight gains than females at respective ages (*p* < 0.05), sex-based averages are not separately presented. Chicks from red LED light-treated eggs had significantly lower live weight at 4 weeks than those in other groups (*p* < 0.05). No significant differences were observed at weeks 6, 10, or 12. However, chicks from the dark group had significantly higher live weight at 8 weeks than those from cold LED light groups (*p* < 0.05). Although this difference was not observed at 12 weeks, at 14 weeks, the red and white LED groups had significantly higher weights than the green group, except for the dark group (*p* < 0.001).

Between weeks 4–6 and 4–8, chicks from red LED and dark groups showed significantly greater weight gains than those from green and white LED groups (*p* < 0.05). From week 4–10, chicks in the red LED group had significantly greater weight gain than the green LED group but were not statistically different from the white and dark groups (*p* < 0.05). From weeks 4–12, weight gains of red and white LED groups were significantly higher than the green LED group. Weight gains of the dark group were not significantly different from others. From weeks 4–14, chicks from the red and white LED groups had significantly higher weight gains than those from other treatments. The green LED group showed the lowest gains, but the dark group was not significantly different from it (*p* < 0.001).

### 3.5. Plasma Corticosterone and Serotonin Hormone Concentrations

**Figure 3:**
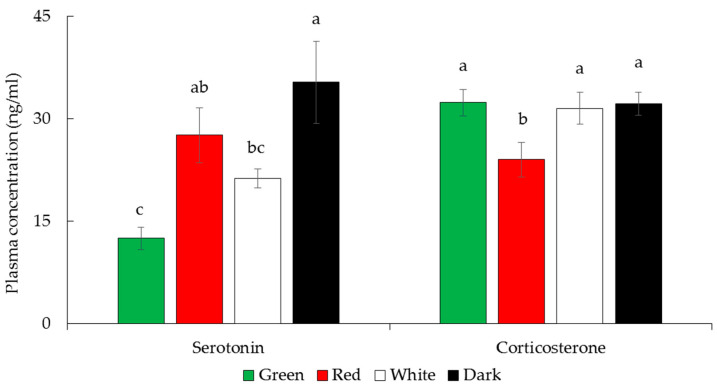
Serotonin and corticosterone levels in treatment groups. ^a,b,c^ Different letters above the bars indicate statistically significant difference at *p* < 0.05.

Significant differences were found in plasma serotonin levels among treatment groups (*p* < 0.05). The highest serotonin concentration was observed in chicks from the dark group (35.38 ± 6.01 ng/mL), followed by red (27.60 ± 4.01 ng/mL), white (21.26 ± 1.37 ng/mL), and green (12.50 ± 1.60 ng/mL) LED groups. Plasma corticosterone levels were also analyzed. No significant differences were found among chicks from green (32.36 ± 1.89 ng/mL), white (31.56 ± 2.31 ng/mL), cold LED (32.22 ± 1.72 ng/mL), and dark (32.22 ± 1.72 ng/mL) groups. However, corticosterone levels were significantly lower in the red LED group (24.03 ± 2.50 ng/mL) compared to all others (Figure 3).

### 3.6. Myogenic Gene Expression

**Figure 4:**
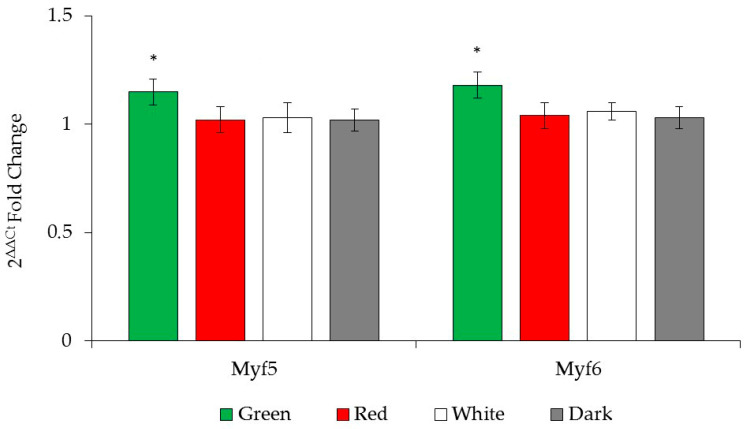
Gene expression levels of myogenic factor 5 (Myf5) and myogenic factor 6 (Myf6) genes in PM and GN muscles of chicks in treatment groups. * *p* = 0.073.

The gene expression levels of Myf5 and Myf6 in PM and GN muscles based on the 2^−∆∆Ct^ fold change method are shown in Figure 4, and the correlations between daily weight gain and gene expression levels up to the 14th week are presented in Table 6. No significant two-way or three-way interactions were observed for group, muscle type, or sex (*p* > 0.05); therefore, only the main effects are provided. Since male and female chicks had similar Myf5 and Myf6 gene expression levels in both PM and GN muscles, these values are not presented separately. Although no statistically significant differences were detected among the treatment groups in terms of Myf5 and Myf6 gene expression levels, chicks from the green LED light group showed a tendency toward higher expression of both genes (*p* = 0.073). Additionally, no significant correlations were found between daily live weight gain and Myf5 or Myf6 gene expression levels up to 14 weeks.

## 4. Discussion

### 4.1. Spectral Modulation of Embryonic Development

The present study systematically investigated the effects of cold LED lights of varying colors applied during the pre-incubation period on hatching time and the hatching window in Gerze chickens. The results revealed that light color significantly influences embryonic development dynamics and markedly alters the hatching process. In particular, the hatching window of embryos exposed to red LED light (42 h) was almost half that of other light treatments (75 h for green and white, 84 h for dark). These findings indicate that red LED light may enhance embryonic developmental rates, thereby advancing hatching onset and reducing the overall duration of the hatching period. One plausible mechanism underlying this phenomenon is that red light increases embryonic metabolic activity, accelerating developmental progression [28,29]. The notably prolonged hatching duration in the absence of light supports the hypothesis that photostimulation facilitates embryonic development and effectively shortens the incubation period. Statistical analyses further corroborate that light color significantly modulates the timing and synchrony of hatching in Gerze chickens. In particular, the markedly reduced hatching window observed under red LED illumination underscores its potential as an effective intervention for optimizing incubation efficiency and synchronizing chick emergence.

Previous studies have identified differences in the responses of genetic lines to different wavelengths of light applied during incubation [30]. It has been reported that green LED light application during incubation accelerates broiler chickens (Arbor Acres) embryonic development [23]. Similar to our findings, Tong et al. [16] reported that the individual incubation period in the green light group was 3.4 h earlier compared to the dark group in Ross 308 broiler chickens. Wang et al. [31] also found that green light (200 lux, 12L:12D) advanced the average incubation time by 5.34 h compared to the dark group, concluding that 12L:12D monochromatic green light application during embryogenesis shortened the incubation period without any adverse effects on post-hatch performance in four strains of layer breeder. However, these studies did not include red light. El-Sabrout and Khalil [32] reported that supplementary yellow LED light during incubation improved chick weight at hatch, vitality, and reduced time to hatch in the Egyptian Fayoumi chickens. Our study suggests that red LED light is more effective in shortening incubation time than green light, but green LED light also shortened incubation compared to the dark group in Turkish Gerze chickens. In conclusion, it was determined that red LED light accelerates embryonic development during incubation in Gerze chickens, shortening the hatching period; white and green LED light, in comparison to red, prolonged the hatching time, and all LED light applications shortened the period compared to dark incubation. These findings point to the potential of using appropriate lighting strategies in commercial incubation systems to synchronize chick hatching and increase incubation efficiency in slow-growth Gerze chicken. Future studies could expand current knowledge by examining the effects of different light intensities or wavelengths on embryonic development in slow-growing chicken breeds such as Gerze in more detail.

### 4.2. Impacts on Hatchability and Early Chick Vitality

In the current study, the effects of different colored LED lights (green, red, white) and dark applications during the pre-incubation period on incubation efficiency and embryo development were evaluated. The findings showed no statistically significant differences in hatchability, fertility rate, substandard mortality rate, or early, mid, and late embryonic mortality rates. Similarly, Archer [5] reported that different colored LED lights and dark applications during pre-incubation in Cobb500 broiler eggs did not affect hatchability, substandard mortality, or embryonic mortality rates. This suggests that light applications may not have a direct effect on specific stages of embryonic development. However, when evaluated in terms of incubation performance, it was found that the color of light the eggs were exposed to had a significant effect. While there was no difference in incubation performance between green and red LED light applications, white LED light significantly reduced hatchability. Consistent with our findings, Hluchý et al. [15] and Archer [7] reported that red light increased hatchability in broiler and layer eggs. In contrast, Archer [5] found that white light improved hatchability in Cobb500 broiler eggs, while green and dark applications had no effect. Our results suggest that white light may have a detrimental effect on embryonic development in Gerze chicken eggs. Possible reasons may include the broad spectrum nature of white light triggering physiological or biochemical processes that negatively affect embryo development. Previous studies have suggested that hatchability may be related to the stimulation of retinal opsins and extra-retinal photoreceptors [33]. On the other hand, green and red LED light application significantly increased the weight of hatched chicks. This effect was more pronounced compared to dark and white LED light applications. This indicates that green and red light may support embryonic development in Gerze chicken eggs and possibly have a positive effect on metabolic processes. Similarly, green and red LED light groups had higher hatch-to-chick conversion rates than dark and white LED light groups, suggesting a positive effect on embryonic development. Chick weight at hatch was affected by lighting applications during incubation, consistent with previous studies reporting weight increase under green light [18,19,20]. In conclusion, based on the study findings, white LED light use during incubation in Gerze chickens may negatively affect hatchability, whereas green or red LED light applications may enhance chick weight and hatch-to-chick conversion rates. These findings suggest that lighting strategies should be carefully selected in incubation processes for slow-growing breeds such as Gerze, and green or red LED lights may be particularly beneficial.

### 4.3. Long-Term Growth Trajectories and Behavioral Implications

In this study, the effects of different lighting conditions applied during the pre-incubation period on the growth performance of Gerze chicks were also investigated. The main objective of the study was to evaluate the effects of different light colors on the body weight gain of the chicks. The findings revealed that green and white LED light exposure, as well as darkness during the pre-incubation period, had a significantly positive impact on live weight gain in the first four weeks after hatching. However, this effect, particularly observed in chicks incubated under green LED light, was found to diminish after the fourth week. The loss of the positive impact after the 8th week in chicks hatched from eggs incubated under green light suggests that the long-term effects of green light may be limited. On the other hand, chicks hatched from eggs incubated under red LED light had the lowest body weights during the first four weeks; however, in subsequent weeks and closer to the 14th week, they were observed to have significantly higher weights compared to those incubated under green LED light. This finding aligns with the results of Drozdová et al. [34], who reported that chicks incubated under red light had higher body weights during the rapid growth phase compared to those exposed to blue light, which is closer in wavelength to green. Drozdová et al. [34] also noted that chickens incubated under red light exhibited more passive behaviors (resting, standing, preening, dust bathing) and fewer active behaviors (walking, foraging, fighting, wing flapping), which could fundamentally contribute to growth enhancement.

In our study, chicks from eggs incubated under white LED light exhibited the most consistent body weight results and were significantly heavier than those from red light incubation during the first four weeks. However, although their body weights increased steadily and were significantly higher than those of the green light group by the 14th week, no significant differences were observed compared to the red light group at that point. These findings are consistent with the final results reported by Ahmad et al. [35], who observed that chicks from eggs incubated under red LED light showed significantly higher weight gain compared to those incubated under white and green LED lights in a study using Japanese quail. While chicks from white LED light incubation showed better body weights than those incubated under green light, chicks from green light exposure had significantly lower body weights overall. Nevertheless, the finding that red LED light during pre-incubation resulted in higher post-hatch body weight gain compared to other groups indicates that red light may have a growth-promoting effect. Although red light is commonly known for its thermoregulatory effects [36], this finding is consistent with other another study suggesting that different light wavelengths can exert distinct biological effects on animal development [37]. It is particularly well understood that red light also demonstrates similar positive effects on live weight gain. Based on these findings, it can be concluded that red LED light exposure improves growth performance in poultry.

The literature suggests that light exposure can influence chicks’ behavioral responses, growth rates, and immune systems. Each light color has a different wavelength and different wavelengths (white, range at 6000–7500 K; red, range at 610–630 nm; and green, range at 515–525 nm) has an effect on embryonic physiology, hormone activity, and therefore its growth rate and development [5]. Newberry et al. [38] and Rault et al. [39] proposed that light intensity, wavelength, and duration can affect chick stress levels. Archer and Mench [6] observed low corticosterone levels and reported that incubation lighting led to a measurable reduction in chicken stress compared to standard dark incubation. Rozenboim et al. [18,33] reported that exposure to different colors of light during incubation can affect the secretion of corticosterone, a vital hormone in growth regulation and stress response for chick embryos. Moreover, light is able to stimulate embryo growth by stimulating blood flow and metabolism, accelerating tissue growth, and improving embryo growth [40]. Different wavelengths of light can affect the metabolic activity level of the embryo and significantly alter the growth rate by changing energy consumption and body reserves [41]. Previous studies have shown that red light can increase the growth speed of chicken embryos, whereas blue light can affect the nervous system’s progress [18]. Red light activates biological pathways such as the PI3K/Akt pathway, which promotes cell growth and survival and stimulates the growth of tissues and organs in a healthy, balanced state [42,43]. Red light promotes cell proliferation and differentiation, and as a result, encourages organ and tissue growth. Also in our study, the fact that corticosterone levels of chicks hatched from eggs exposed to red LED light were found to be significantly lower at the time of slaughter compared to chicks exposed to other light conditions or darkness may explain the greater weight gain observed between 4 and 14 weeks of age. These findings of the current study reflect the stress-reducing effect of red LED light and confirm its usefulness in improving poultry welfare [34,36]. On the other hand, chicks hatched from eggs incubated in darkness exhibited significantly higher serotonin levels compared to those from light-exposed groups, while those from green LED light incubation had the lowest serotonin levels. This trend in stress hormone variation may be linked to the findings of Drozdová et al. [34], who observed that short-wavelength lights such as blue and green led to more active behaviors in poultry, such as walking, foraging, fighting, and wing flapping. Additionally, even though there was no significant difference in stocking densities among the cages, the variation in group sizes between red and green LED groups may have had an indirect effect on stress levels.

### 4.4. Molecular Regulation of Muscle Development

This study also examined the effects of different colored LED lighting during the incubation period of Gerze eggs on the expression levels of myogenic markers in the PM and GN skeletal muscles of chicks. The findings showed no statistically significant differences in Myf5 and Myf6 gene expression levels; however, green LED exposure tended to result in relatively higher expression levels of both genes. This tendency may be a compensatory response to stress in chicks from green LED-exposed eggs, potentially supporting muscle development. The increase in Myf5 and Myf6 expression in the green LED group may not be entirely attributed to green light exposure alone. Previous studies have also reported that light environment during incubation and early post-embryonic development can influence the physiological and biochemical responses of poultry [30]. It has been suggested that green and blue LED lights can affect metabolic activity and muscle tissue growth rates throughout embryonic development [18]. However, red light was not used in Rozenboim et al.’s study [18]. The effects of green LED light exposure were evident both in hatch weight and in the body weight during the first four weeks. Moreover, the tendency of green LED to increase Myf5 and Myf6 expression levels, although not statistically significant, supports this hypothesis.

Myogenic transcription factors Myf5 and Myf6 play a critical role in skeletal muscle formation and development [44]. Myf5 maintains muscle precursor cell populations, while Myf6 is involved in the differentiation and maturation of muscle cells [45]. Therefore, the trend of increased Myf5 and Myf6 expression under green LED exposure, as shown by higher early body weights, may suggest that early muscle development mechanisms are potentially sensitive to light wavelengths. At the same time, it is likely that other variables—such as nutrition, genetic factors, and environmental conditions, particularly stress—may play a more decisive role in live weight gain. In conclusion, this study revealed that green LED light may have a potential effect on the expression levels of myogenic genes, although this effect did not reach statistical significance. Additionally, the influence of different light environments on long-term growth performance was not clearly evident. Therefore, future studies with larger sample sizes and more detailed molecular analyses are needed to better understand the effects of different light wavelengths on muscle development biology.

## 5. Conclusions

The findings of this present research have several practical implications. The use of red LED light during pre-incubation may be advantageous for optimizing hatchability and shortening the hatch window in the Gerze breed. Green LED light, with its positive effect on initial chick weight, may enhance early chick viability, which is particularly relevant for improving post-hatch survival in slow-growing native breeds. The sustained growth performance associated with red light exposure during the 4–14-week period highlights its potential for improving meat production outcomes and reducing physiological stress, making it especially valuable for longer-term rearing strategies. In contrast, green light may be more beneficial for faster-growing broilers with shorter production cycles.

## Figures and Tables

**Table 1:** Pasgar score quality evaluation criteria [27].

Category	Criteria for Scoring
Reflex	Chicks take more than 2 s to return to normal position when flipped onto their back
Navel	Abnormalities such as small white or black button-shaped belly, yellow residue, or open navel
Legs	Red hocks, swollen legs, or leg deformities
Beak	Red spots, egg white contamination on the nostrils, or deformities
Abdomen	Complete exhaustion or remaining hardness from egg yolk

**Table 2:** Primers used in qPCR analysis.

Genes	Primer Sequences (5′-3′)	Product Size (bp)	Gen Bank
Mfy5-F	CGGAAGGCAGCCACTATGAG	150	NM_001030363
Mfy5-R	GAGGCTTTCGATGTACCTGATG		
Mfy6-F	GCCTGCAAAACCTGCAAGAG	150	NM_001030746
Mfy6-R	AGTCCGCCTTTTCAGAGCCT		
GAPDH-F	CTGCCCAGAACATCATCCCA	139	K01458
GAPDH-R	CGGCAGGTCAGGTCAACAAC		

**Table 3:** Average initial weights of eggs in the treatment group, storage loss, and weight change during incubation.

Parameters	Group				*p*
	Green	Red	White	Dark	
EN	114	114	114	108	1000
EIW (g)	59.068 ± 0.302	59.394 ± 0.347	58.691 ± 0.323	58.241 ± 0.332	0.540
ETW (g)	52.901 ± 0.301	53.505 ± 0.318	52.390 ± 0.314	52.010 ± 0.343	0.928
ESL (g)	6.167 ± 0.078	5.889 ± 0.122	6.301 ± 0.119	6.231 ± 0.082	0.125

EN = egg number; EIW = egg initial weight; ETW = egg transfer weight; ESL = egg storage loss.

**Table 4:** Hatching characteristics of eggs in the treatment group and average post-hatching Pasgar scores of chicks.

Parameters	Group				*p*
	Green	Red	White	Dark	
HE (%)	87.72 ^a^	81.58 ^a^	78.07 ^b^	79.63 ^a^	0.042
H (%)	90.91	86.92	79.46	84.31	0.739
F (%)	110.00	107	98.25	94.44	0.999
UD (%)	0.00	0.00	2.68	0.00	0.666
EED (%)	0.00	1.87	0.00	0.00	0.548
MED (%)	0.91	0.00	0.00	1.96	0.681
LED (%)	8.18	11.21	17.86	13.73	0.129
TED (%)	9.09	13.08	17.86	15.69	0.096
CW (g)	40.86 ± 0.29 ^a^	41.98 ± 0.34 ^a^	40.28 ± 0.32 ^b^	40.17 ± 0.36 ^b^	<0.001
ECR (%)	69.17 ^a^	70.62 ^a^	68.63 ^b^	68.97 ^b^	<0.001
PS	9.78 ± 0.53	9.70 ± 0.05	9.57 ± 0.07	9.56 ± 0.08	0.516

^a,b^ The differences between the means shown with different letters in the same row are significant (*p* < 0.05, *p* < 0.001). HE = hatching efficiency; H = hatchability; F = fertility; UD = undershell death; EED = early embryonic death; MED = middle embryonic death; LED = late embryonic death; TED = total embryonic death; CW = chick weight; ECR = egg to chick conversion rate; PS = Pasgar score.

**Table 5:** Live weights and live weight gains of chicks in the treatment group at various ages.

Parameter (g)	Group				*p*
	Green	Red	White	Dark	
	n = 81	n = 62	n = 69	n = 70	
4.W	304.52 ± 4.97 ^a^	283.03 ± 5.27 ^b^	301.61 ± 5.73 ^a^	309.93 ± 7.10 ^a^	0.009
6.W	436.17 ± 6.78	440.65 ± 8.33	424.77 ± 7.76	448.69 ± 8.86	0.653
8.W	601.99 ± 9.82 ^b^	611.90 ± 12.00 ^b^	605.10 ± 10.20 ^b^	648.10 ± 12.20 ^a^	0.031
10.W	796.50 ± 13.30	825.60 ± 15.90	823.20 ± 14.80	840.40 ± 15.80	0.660
12.W	956.10 ± 15.50	1014.80 ± 20.00	1019.30 ± 18.70	1004.50 ± 18.40	0.545
14.W	1060.30 ± 18.10 ^b^	1196.90 ± 24.00 ^a^	1182.60 ± 22.90 ^a^	1126.20 ± 22.50 ^a,b^	<0.001
4–6W	131.65 ± 3.67 ^b^	157.61 ± 4.30 ^a^	123.14 ± 4.60 ^b^	138.76 ± 10.40 ^a^	0.002
4–8W LWG	297.47 ± 6.13 ^b^	328.89 ± 8.23 ^a^	303.82 ± 6.37 ^b^	338.20 ± 13.20 ^a^	0.002
4–10W LWG	492.00 ± 10.10 ^b^	542.60 ± 12.40 ^a^	522.70 ± 10.90 ^a,b^	530.50 ± 16.50 ^a,b^	0.029
4–12W LWG	651.60 ± 12.7 ^b^	731.80 ± 17.00 ^a^	718.90 ± 15.10 ^a^	694.50 ± 18.90 ^a,b^	0.002
4–14 W LWG	755.80 ± 16.00 ^c^	913.80 ± 21.70 ^a^	880.70 ± 19.30 ^a,b^	816.30 ± 22.90 ^b,c^	<0.001

^a,b,c^ The differences between the means shown with different letters in the same line are significant (*p* < 0.05, *p* < 0.001). W = week; LWG = live weight gain.

**Table 6:** Chicks in the treatment group 4–14W correlation coefficients between daily live weight gain and expression levels of Myf5 and Myf6 genes.

	Green	Red	White	Darkness	*p*
	DLWG				
Myf5	0.329	0.129	0.146	0.207	0.214
Myf6	0.418	0.158	0.163	0.143	0.117

DLWG = 4–14th week daily live weight gain in weekly age period.

## Data Availability

Data presented in this study are available on request from the corresponding author.
